# The cylindromatosis (*CYLD*) gene and head and neck tumorigenesis

**DOI:** 10.1186/s41199-016-0012-y

**Published:** 2016-09-08

**Authors:** Krista Roberta Verhoeft, Hoi Lam Ngan, Vivian Wai Yan Lui

**Affiliations:** 1 Department of Clinical Oncology, Li-Ka Shing Faculty of Medicine, the University of Hong Kong, Hongkong, SAR Hong Kong; 2 School of Biomedical Sciences, Li-Ka Shing Faculty of Medicine, the University of Hong Kong, Hongkong, SAR Hong Kong; 3 School of Biomedical Sciences, Faculty of Medicine, the Chinese University of Hong Kong, Hongkong, SAR Hong Kong

**Keywords:** Head and Neck Cancer, Cylindromatosis (*CYLD*), The *CYLD* cutaneous syndrome, Turban Tumor Syndrome, Brooke-Spiegler Syndrome (BSS), Multiple Familial Trichoepithelioma (MFT1), Familial Cylindromatosis (FC), tumorigenesis, Deubiquitinating (DUB), Nuclear Factor-kB (NF-kB), TNF-receptor associated factor (TRAF) proteins, and B-cell lymphoma 3 (Bcl-3)

## Abstract

Germline *CYLD* mutation is associated with the development of a rare inheritable syndrome, called the *CYLD* cutaneous syndrome. Patients with this syndrome are distinctly presented with multiple tumors in the head and neck region, which can grow in size and number over time. Some of these benign head and neck tumors can turn into malignancies in some individuals. *CYLD* has been identified to be the only tumor suppressor gene reported to be associated with this syndrome thus far. Here, we summarize all reported *CYLD* germline mutations associated with this syndrome, as well as the reported paired somatic *CYLD* mutations of the developed tumors. Interestingly, whole-exome sequencing (WES) studies of multiple cancer types also revealed *CYLD* mutations in many human malignancies, including head and neck cancers and several epithelial cancers. Currently, the role of *CYLD* mutations in head and neck carcinogenesis and other cancers is poorly defined. We hope that this timely review of recent findings on *CYLD* genetics and animal models for oncogenesis can provide important insights into the mechanism of head and neck tumorigenesis.

**Electronic supplementary material:**

The online version of this article (doi:10.1186/s41199-016-0012-y) contains supplementary material, which is available to authorized users.

## Introduction

Understanding of genetic diseases that are closely linked to tumor development can provide important insights into the biology of human tumorigenesis and treatment. To date, only a handful of human genetic diseases are uniquely associated with predisposition of head and neck tumor formation. In this focused review, we will provide an up-to-date summary of the cylindromatosis (*CYLD*) gene defects in a genetic disease called the *CYLD* cutaneous syndrome. This genetic syndrome is, in particular, characterized by multiple tumor formation in the head and neck region often with early age onset. Some of these tumors will remain benign, while some can turn malignant. Interestingly, *CYLD* genetic aberrations have recently been reported by recent whole-exome sequencing (WES) studies in head and neck cancers, and some other cancers, thus revealing its potential involvement in human carcinogenesis. Therefore, it is timely to review the genomic aberrations of *CYLD* in this particular genetic disease, which will deepen our understanding of human tumorigenesis, in particular, of the head and neck.

### The *CYLD* gene

The *CYLD* gene (chr 16q12.1) codes for a 107 kDa cytoplasmic deubiquitinating (DUB) enzyme, which removes ubiquitin molecules from various signaling proteins, and regulates the activities of many cellular and signaling processes. This gene was first discovered and cloned in 2000 by Bignell et al. with prior evidence suggesting the existence of a potential tumor suppressor gene on chr 16q12-q13 linked to a peculiar cutaneous disease characterized by multiple tumors in the head and neck region [1]. Subsequent functional studies revealed multiple roles of CYLD in the regulation of inflammation, immunity, cell cycle progression, spermatogenesis, osteoclastogenesis, ciliogenesis, migration and potentially tumorigenesis [1–4]. To date, several major signaling pathways have been found to be linked with or regulated by CYLD, which include the Nuclear Factor-kB (NF-kB), Wnt/β-catenin and c-Jun NH(2)-terminal kinase (JNK) pathways, and potentially others [5–7]. Genetic alterations of *CYLD* could result in aberrant activation or inhibition of these signaling pathways, which may contribute to disease pathology.

### The CYLD cutaneous syndrome

In 1842, a rare cutaneous disease was first described in a female patient, named Frances Massenger, who developed multiple tumors in the head, neck and face. In addition to her early disease onset at age 14, multiple family members of this patient also had a history of head and neck tumors [8], which strongly implied a potential underlying genetic cause of this rare disease. Over a century later in 1995, Biggs et al. discovered the locus of the susceptibility gene on chromosome 16q12-q13 by linkage analysis of the members of two affected families, revealing the potential loss of a likely tumor suppressive gene associated with this rare syndrome [9]. The following year, Biggs et al. provided further evidence to suggest that *CYLD* (referred to as *Cyld1*) may be the only tumor suppressor gene involved in the *CYLD* cutaneous syndrome [10]. A subsequent larger study with 21 affected families ultimately helped to identify the gene associated with this syndrome to be the *CYLD* gene on chromosome 16q12 and detected, for the first time, germline and somatic mutations of *CYLD* in affected patients [1]. The gene was cloned by fine-mapping and positional cloning and it was confirmed that *CYLD* germline mutations are associated with and are the underlying cause of this cutaneous syndrome in humans [1].

The term, *CYLD* cutaneous syndrome, was proposed recently by Rajan et al. [11] to describe this rare inheritable condition that is known to be caused by germline mutations of the *CYLD* gene based on genetic evidence [9]. The occurrence rate of *CYLD* germline defects is ~1:100,000 based on the UK data [12]. Patients with this syndrome are clinically characterized with multiple tumors of the skin appendages often in the head and neck region (i.e. skin lesions derived from the epidermal appendages, hair follicles, sweat apparatus, etc.). The *CYLD* syndrome encompasses three previously known appendageal tumor predisposition syndromes: familial cylindromatosis (FC, or Turban tumor syndrome; OMIM 132700), multiple familial trichoepithelioma 1 (MFT1; also called epithelioma adenoides cysticum, EAC, or Brooke-Fordyce trichoepitheliomas; OMIM 601606), and Brooke-Spiegler syndrome (BSS or BRSS; OMIM 605041), which are believed to be allelic disorders with overlapping phenotypes associated with *CYLD* mutations. The clinical manifestations of these *CYLD-*associated syndromes as well as the images for the head and neck, and facial manifestations have been recently reviewed [13]. All three tumor predisposition syndromes are autosomal dominant disorders, in which a germline *CYLD* mutation was inherited, and a second, non-inherited *CYLD* mutation or loss of heterozygosity (LOH) occurs in cells for tumor formation. FC is typically presented with multiple cylindromas (i.e. benign tumors with differentiation towards apocrine sweat glands that increase in number and size over age). These multiple cylindromas growing in the scalp may coalesce and cover the entire scalp like a turban (thus FC is also called the Turban tumor syndrome). MFT1 is characterized by multiple trichoepitheliomas (i.e. skin tumors on the face with histologic dermal aggregates of basaloid cells with connection to or differentiation toward hair follicles), which can turn into basal cell carcinoma [14]. BSS, mostly with early adulthood onset, is classically characterized by multiple skin appendage tumors including cylindroma, trichoepithelioma, and spiradenoma (eccrine spiradenomas or cystic epitheliomas of the sweat gland, usually solitary, deep-seated dermal nodule typically located in the head and neck region [15]). Since members of a single family can manifest as FC, MFT1 or BSS with *CYLD* aberrations, many consider these three diseases as a phenotypic spectrum of a single disease entity with underlying *CYLD* mutation. These tumors can be painful, itchy and irritating, and in some cases, turn to malignancies. Due to the very disfiguring nature of these head and neck, facial tumors, surgical removal and often repeated surgeries are performed on these individuals to limit tumor growth over their life-time. The psychological impacts due to the disfiguring appearance of affected individuals may lead to depression and social withdrawal [16].

To date, the *CYLD* cutaneous syndrome has been reported in various ethnic backgrounds, with age onset as early as 5, to 40 years old. The average age onset is around teenage (~16 years old) [11]. Such an early age onset of multiple tumor formation distinctly in the head and neck region strongly imply a potential critical role of *CYLD* mutations in promoting head and neck tumorigenesis.

### *CYLD* Germline and somatic mutations in individuals with the CYLD cutaneous syndrome

**Fig. 1:**
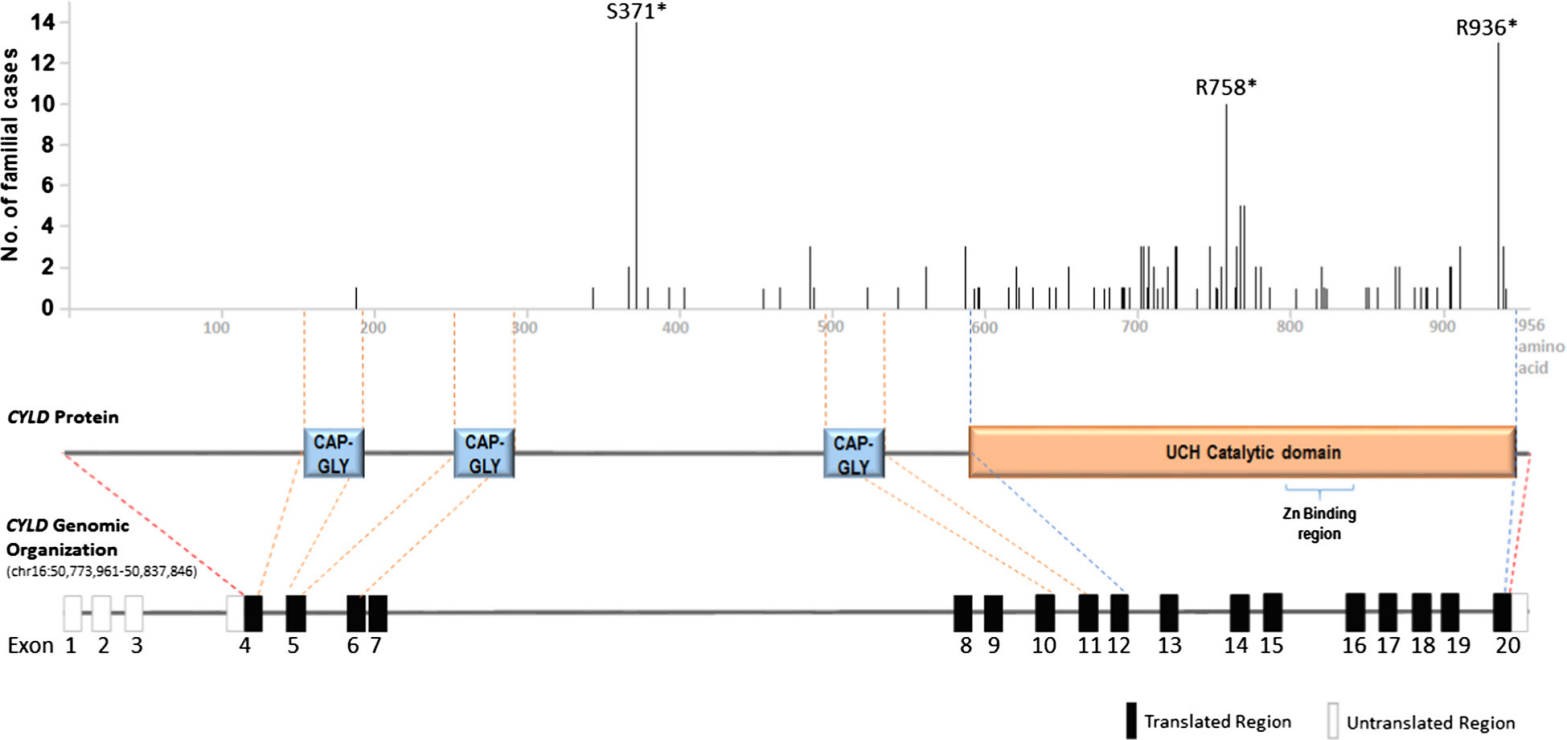
Reported *CYLD* germline mutations in patients with the *CYLD* cutaneous syndrome [1, 11, 17, 19–23, 25, 26, 81, 120]. The frequency of familial cases of *CYLD* cutaneous syndrome with germline *CYLD* mutations, and the corresponding amino acid positions affected by these mutations are indicated (as detailed in Table 1 and predicted using the Integrative Genomics Viewer (IGV) software, the Broad Institute, USA). The CYLD protein contains three CAP-GLY domains (aa 155–198, 253–286, 492–535), a UCH catalytic domain (aa 591–950) and a Zinc binding region (aa 778–842) within in the catalytic domain based on the NCBI number NP_056062.1

As of today, a total of 107 germline *CYLD* mutations have been reported in patients developing FC, BSS and MFT1 (Table 1). Most reported mutations reside between exons 9 and 20 of the *CYLD* gene. The current data revealed several hotspot mutation sites of *CYLD*: 1112C > A (S371*), 2272C > T (R758*) and 2806C > T (R936*) in 14, 10 and 13 independent families, respectively [17–19] (Fig. 1). Note that all three hotspot mutations are nonsense mutations, which are likely to produce truncated forms of the CYLD protein, potentially representing loss-of-function of the CYLD protein. In fact, the majority of *CYLD* germline mutations are deleterious mutations, including frameshift (44 %), splice-site (11 %), nonsense mutations (25 %), germline deletions (2.7 %) followed by missense mutations (11 %) and silent mutations (1 %) (Table 1). Note that a few studies reported the absence of detectable *CYLD* germline mutation in a small number of affected individuals [20, 21]. It is possible that some *CYLD* alterations may have been missed as these previous studies examined only certain exons/regions *CYLD* using direct sequencing, or probe-based fluorescence in-situ hybridization (FISH) or linkage analysis. Thus far, no single study has sequenced the entire *CYLD* gene including the regulatory and intronic regions, which can also be potentially altered but missed by targeted sequencing. Note that sporadic occurrences of the syndrome have also been reported. In those cases, only the affected individual, but not their family members, will carry a germline *CYLD* mutation and present with the syndrome phenotype [22, 23].

**Table 1:** Germline *CYLD* mutations reported in patients with the *CYLD* cutaneous syndrome

Exon	Germline *CYLD* mutations		No. of families	Reference
	DNA	Protein		
5	561-562dupT	Q188Sfs	1	[23]
9	1027dupA	T343Nfs	1	[20]
9	1096_1097delCA	Q366Tfs	2	[20, 81]
9	1112C > A	S371^a^	14	[1, 17, 20, 21, 81–85]
9	1135G > T	E379^a^	1	[86, 87]
10	1139-1148A > G	splice site mutation	1	[20]
10	1178_1179delCA	*T393Rfs*	1	[88]
10	1207C > T	*Q403* ^a^	1	[86, 87]
10	1364_1365delAA	Q455Rfs	1	[89]
10	1392_1393dupT	G465Wfs	1	[23]
10	1455 T > G	Y485^a^	1	[90]
10	1455 T > A	Y485^a^	2	[1, 20]
10	1462delA	I488Sfs	1	[25]
10	1473C > T	*I491I*	unavailable	[91]
10	1518 + 2 T > C	*splice site mutation*	1	[92]
11	1569 T > G	Y523^a^	1	[1]
11	1628del2	S543^a^	1	[81]
11	1681_1682del	L561Sfs	1	[1]
11	1682 T > A	L561^a^	1	[85]
11	1684 + 1G > A	splice site mutation	2	[20, 93]
12	1758insGATA	M587Dfs	2	[20, 82]
12	1758ins2	*M587fs*	1	[81]
12	1776delA	G593Afs	1	[1]
12	1783C > T	*Q595* ^a^	1	[94]
12	1787G > A	G596D	1	[95]
12	1821_1826 + 1del-insCT	*splice site mutation*	1	[96]
12	1826 + 1G > A	splice site mutation	1	[97]
12	1826 + 1G > T	splice site mutation	2	[20, 21]
13	1830-1831insA	*F611Ifs*	1	[1]
13	1843delT	*S615Lfs*	1	[98]
13	1859_1860delTG	V620fs	2	[1, 81]
13	1863insA	L622Tfs	1	[81]
13	1893_1906delATATTATAGTGAAA	*E631Dfs*	1	[23]
13	1925delC	*T642Kfs*	1	[108]
13	1935dupT	N646^a^	1	[1]
14	1950-2A > T	*splice site mutation*	1	[23]
14	1950_1953-1delGATA	*splice site mutation*	1	[23]
14	1961 T > A	V654E	2	[26]
14	2012-2021del10	A671Dfs	1	[99]
14	2032G > T	*E678* ^a^	unavailable	[91]
15	2041 + 1G > T	splice site	1	[100]
15	2042-1G > C	splice site	1	[109]
15	2042A > G	D681G	1	[86, 87]
15	2065_2066delCT	*L689Vfs*	1	[85]
15	2068_2069delTTinsC	*F690Lfs*	1	[85]
15	2070delT	*H691Ifs*	1	[110]
15	2081delT	L694^a^	1	[86, 87]
15	2104delA	I702^a^	2	[20, 90]
15	2104_2105insA	I702Nfs	1	[24]
15	2108G > A	R703K	1	[111]
15	2108G > C	R703T	2	[20, 90]
16	2116_2117insATTAG	G706Dfs	1	[112]
16	2119C > T	Q707^a^	3	[20, 90]
16	2128C > T	*Q710* ^a^	2	[25, 108]
16	2138delA	Y713Sfs	1	[1]
16	2146C > A	*Q716K*	1	[85]
16	2154insT	*M719Yfs*	1	[81]
16	2155dupA	M719Nfs	1	[20]
16	2170_2172insTC	K724Ifs	3	[20, 90]
16	2172delA	V725Lfs	3	[1, 15, 81]
16	2214delT	F738Lfs	1	[81]
16	2240A > G	E747G	2	[81, 101]
16	2240_2241delAG	E747fs	1	[102]
17	2252delG	C751Ffs	1	[103]
17	2255delT	*L752Rfs*	1	[83]
17	2259dupT	I754Yfs	2	[20, 21]
17	2272C > T	R758^a^	10	[1, 18, 20, 21, 85, 104, 113]
17	2288_2289delTT	F763^a^	1	[20]
17	2290_2294del	K764Ifs	1	[81]
17	2291_2295delAACTA	K764Ifs	2	[20]
17	2299A > T	K767^a^	5	[20, 83, 90]
17	2305_2306insC	I769Tfs	1	[1]
17	2305delA	I769Ffs	4	[82]
17	2330_2331delTA	I777Nfs	2	[20, 105]
17	2339 T > G	L780^a^	2	[81, 82]
18	2350 + 5G > A	Splice Site Mutation	2	[1, 85]
18	2355_2358delCAGA	R786Sfs	1	[106]
18	2409C > G	Y803^a^	1	[89]
18	2449delT	C817Vfs	1	[114]
18	2460delC	*C820* ^a^	2	[1, 11]
18	2465insAACA	*T822Tfs*	1	[107]
18	2467C > T	Q823^a^	1	[1]
18	2469 + 26G > A	splice site mutation	1	[99]
18	2469 + 1G > A	splice site mutation	2	[1, 11]
19	2546G > A	W849^a^	1	[86, 87]
19	2552_2553insA	*H851Qfs*	1	[115]
19	2569C > T	Q857^a^	1	[1]
19	2602G > T	E868^a^	2	[1, 116]
19	2613C > G	H871Q^#^	2	[91, 117]
19	2641delG	D881Tfs	1	[20]
19	2655G > A	*W885* ^a^	1	[85]
19	2662_2664delTTT	*F888del*	1	[85]
19	2666A > T	D889V	1	[96]
20	2687G > C	G896A	1	[118]
20	2709dupT	*P904Sfs*	1	[119]
20	2711C > T	P904L	1	[83]
20	2712delT	*Q905Kfs*	1	[96]
20	2713C > T	Q905^a^	1	[20]
20	2729dupC	E911Rfs	3	[20, 90]
20	2806C > T	R936^a^	13	[1, 19, 20, 22, 26, 81, 82, 91, 120]
20	2814_2817delGCTT	L939Vfs	3	[20, 90]
20	2822A > T	D941V	1	[25]
-	2686 + 60_^a^3340del5632^b^	germline deletion	1	[85]
-	34111_^a^297858del378779^c^	germline deletion	1	[121]
-	914-6398_1769del13642ins20^d^	germline deletion	1	[121]

A total of 107 germline mutations of *CYLD* have been reported in the literature thus far. This table summarizes the reported DNA changes, protein changes, frequency and original report of 105 germline *CYLD* mutations. Two additional germline mutations of *CYLD* were originally reported as 1862 + 2 T > G (splice site mutation) [102] and 2317G > A [122], however, the protein change cannot be interpreted by sequence analysis and are therefore not included in this table. Based on the nucleotide sequences provided by the original articles, we predicted the mutational changes on the CYLD protein using the Integrated Genomic Viewer (IGV) software (Broad Institute, USA) as *italicized*- based on the reference GenBank number NM_015247 for *CYLD*. Abbreviations: *del* deletion, *ins* insertion, *dup* duplicate, ^a^ = introduction of stop codon. Notes: ^b^Large deletion (~5.3kB) in the catalytic domain UCH region of *CYLD*. ^c^Large deletion (~13.6kB) from intron 6 to exon 12 affecting the 3rd CAP domain and beginning of the UCH domain, additionally, a 20 bp insertion was detected. ^d^Large deletion (0.4 MB) of entire *CYLD* gene and some surrounding regions

Theoretically, it is possible that other genetic events, besides *CYLD*, may be involved. Candidates like Patched 1 (*PTCH1*) has been proposed earlier, but later disputed to be a potential candidate for the *CYLD* cutaneous syndrome [21, 24, 25]. As next-generation sequencing (NGS) can now be easily employed to study various diseases, it is likely that whole-exome or even whole-genome studies of these head and neck tumors from affected individuals can reveal previously unidentified genetic changes associated with the disease, in addition to *CYLD*.

Patients with the *CYLD* cutaneous syndrome inherit one copy of the mutated *CYLD* gene, while LOH or mutation of the second copy of the *CYLD* gene occur somatically for tumor formation. Several studies investigated the actual genetic change of *CYLD* in the developed tumors versus that of the germline aberrations in affected individuals. A total of 15 such cases have been reported thus far. As shown in Table 2, tumors from each of the 15 cases all harbored additional *CYLD* aberration(s) different from the original germline *CYLD* mutation. In some cases, somatic *CYLD* changes among different tumors of the same individual can also be different. In general, nonsense *CYLD* mutations seem to be the most common germline event, while LOH or loss-of function *CYLD* mutations (nonsense, or frameshift mutations) were frequently detected as somatic events (Table 2). This genetic pattern is supportive of the 2-hit hypothesis of tumorigenesis, similar to that of the retinoblastoma 1 (*RB1*) gene alterations for the development of retinoblastoma. Not only genetic heterogeneity was observed among tumors from the same individual, the pathologies of these tumors can also vary from benign to malignant in some cases. It is likely that *CYLD* alteration is an early event for head and neck tumorigenesis, and potentially supportive of later malignant transformation over time.

**Table 2:** Reported paired germline and somatic *CYLD* mutations in patients with the *CYLD* cutaneous syndrome

Age of onset, Gender	Severity	Germline Mutation			Somatic Mutation		Malignancy	Sequencing method and reference
		DNA	Protein		DNA	Protein		
35, F	Md	2070delT	F690fs	T1 T2 T3	n.s., n.s. n.s. undetectable	I645V, R936^c^ Q731^c^ undetectable	Benign Benign Benign	PCR. Sequenced *CYLD* coding regions (exons 4–20) and splice sites. GenBank#:NT010498.15 [110]
24, F	S-VS	2806C > T	R936^c^	T1	n.s., n.s.	R936^c^, D889N	Benign	PCR. All *CYLD* exons [22]
teens, F	S	2012-2021del 10, 2469 + 26G > A	A671fs, splice site mutation	T1	LOH	-	BCC	PCR. Sequenced *CYLD* coding regions and splice sites. GenBank#: NT010505. Tumor LOH analysis using markers:D16S3044, D16S308, D16S503 [99]
n.s., M	Md	2104_2105insA	I702fs	T1	2541G > A	W847^c^	Benign	PCR. Sequenced *CYLD* coding regions (exons 4–19) and splice site. GenBank#:AJ250014. Tumor LOH analysis using markers: D9S925, D9S171 & D9S169-(chr.9p2), D9S15, D9S252, D9S303, and D9S287 (chr.9q22.3) and D16S 769, D16S 753, CDRP 28, CDRP 23, D16S 416, D16S 771, D16S 673 (chr.16) [24]
n.s., F (Family1 mother)	S	1455 T > G	Y458^c^	T1 T2 T3	1736_1739dupTGGA LOH 1794C > A	E580Dfs - Y598^c^	n.s.	Sequenced *CYLD* coding and non-coding regions (exons 1–20) were analyzed. GenBank#:AC007728. Tumor LOH analysis using markers: D16S304, D16S308, D16S419, D16S476, and D16S541 (chr.16q) and D16S407 (chr.16p) [90]
n.s., F (Family1 daughter)	S	1455 T > G	Y458^c^	T1 T2 T3	LOH LOH 1540dupA	- - T514Nfs	n.s. n.s. n.s.	
n.s., F (Family2 mother)	S	2104delA	I702^c^	T1	1112C > A	S371^c^	n.s.	
n.s., F (Family2 daughter)	Md-S	2104delA	I702^c^	T1	2467C > T	Q823^c^	n.s.	
n.s., M	S	2108G > C	R703T	T1 T2	2806C > T LOH	R936^c^ -	n.s. n.s.	
n.s., F	Md-S	2119C > T	Q707^c^	T1 T2	LOH 2713C > T	- Q905^c^	n.s. n.s.	
46, F	Md	2170_2171insTC	K724Ifs	T1	2046_2047ins AGATCCG	E683Rfs	n.s.	
18, F	S	2299A > T 2279dupC 2279dupC	K767^c^ E911Rfs E911Rfs	T1 T2 T3	LOH LOH 2107A > T	- - R703^c^	n.s.	
n.s., F	Md-S	2729dup C	E911Ffs	T1	LOH	-	n.s.	
n.s., M	Md-S	2814_2817delGCTT	L939Vfs	T1	LOH	-	n.s.	
~26, M	S	1684 + 1G > A	splice site mutation	T1T2 T3 T4 T5	LOH LOH LOH LOH 2322delA	- - - - E774Dfs	Benign Benign BCC BCC CCD	Sequencing regions were not reported. Tumor LOH analysis was performed (markers not specified) [93]

The *CYLD* cutaneous syndrome patient cases reported with paired germline and somatic *CYLD* mutations; and including disease severity information and reported sequencing methods. Severity was defined as mild (Md), severe (S) or very severe (VS) using the following criteria: Md = few, small tumors, not painful or overgrowing. S = Multiple large growths, painful/ulcerating and resulting in tumor excision. VS = Multiple large tumors, often disfiguring, painful/ulcerating, resulting in multiple tumor excisions and/or complete scalp removal. Abbreviations: *del* deletion, *ins* insertion, *dup* duplicate, ^c^ = introduction of stop codon, *BCC* basal cell carcinoma, *LOH* Loss of heterozygosity, *n.s*. not stated, *CCD* clear cell differentiation, *T* individual tumor used for analysis

Notes: ^a^Cases from related members of a family (mother and daughter), ^b^Cases from another family (mother and daughter)

### *CYLD* aberrations with benign tumor formation or malignant transformation?

Most clinical reports on the *CYLD* cutaneous syndrome indicate that the majority of tumors developed in the head and neck region are benign in nature, with progressive growth in size and number over one’s lifetime. However, emerging evidence is supportive of malignant transformation of these usually benign tumors into malignancies in some affected individuals, perhaps even in situ, arising from the original benign tumors [26]. In fact, the very first case report of such cutaneous syndrome (though with unclear genetics), had extensively documented multiple tumor formation in the patient's peritoneum, reminiscent of the patient’s head and neck tumors. The patient who later manifested a state of cachexia did suggest a “malignancy” as indicated in the report [8]. Yet, it remains unclear if these tumors in the peritoneum were originated in situ or were actually metastatic lesions from the head and neck tumors.

Due to the rarity of the syndrome, and repeated surgeries for most patients (for cosmetic reasons), documentation of malignant transformation of these seemingly benign tumors is scarce. Recently, Kazakov et al. reported multiple cases with histological evidences suggesting that the malignant lesions seemed to develop or transform in situ at the original “benign” tumors of the cutaneous syndrome patients [26]. A histological study showed that in an invasive carcinoma, the basal cell adenocarcinoma (BCAC) of the salivary gland that was developed in the affected individual, there remained a residuum of spiradenoma which merged with the invasive carcinoma by histology. Similar findings in another affected individual showed that the benign tumor had developed into an invasive lesion in the skull with a BCAC histology. Invasive adenomas of various histologies have been identified in several affected individuals as well. How did these malignant transformations occur in situ? Did the tumors acquire additional genetic aberrations that caused or supported malignant transformation? Or were the *CYLD* genetic aberrations (two copies of *CYLD* mutated or loss) sufficient to drive such a malignant transformation over time if the tumors had not been excised early enough by surgery?

As demonstrated by chemically-induced colon and liver cancer models with *CYLD* ^*−/−*^ mice [16, 27], it seems that phenotypically invasive or potentially metastatic tumors can develop with a *CYLD* deficient background in vivo. This may imply that *CYLD* loss, together with a strong cancer inducing agent or DNA mutagen, can turn normal cells to tumors with the potential to further transform into malignancies. This notion is further supported by findings from Alameda et al. that expression of a catalytically inactive form of CYLD in a Ha-*ras*-mutated tumorigenic epidermal cell line (PDVC57) significantly promoted in vitro cell proliferation, migration (with changes to a mesenchymal phenotype), anchorage-independent growth, as well as pronounced in vivo tumor growth and angiogenesis with upregulation of vascular endothelial growth factor-A (VEGF-A) expression [28]. Using a subcutaneous tumor model, the authors demonstrated that the *CYLD* mutant tumors not only grew faster and larger in size, but also showed a more aggressive, poorly differentiated phenotype when compared to the control tumors which bore a less aggressive, differentiated phenotype. It was hypothesized that the presence of Ha-*ras* mutation in this cell model, PDVC57, together with *CYLD* mutation, may be responsible for such an aggressive phenotype, which is in contrast with the observed benign skin tumors developed in *CYLD* ^*−/−*^ mice as previously reported by Massoumi et al. [29]. These findings may suggest that CYLD may cooperate with other oncogenic events, in this case Ha-*ras* mutation, to promote malignant transformation. Thus, future investigations on *CYLD* gene interaction may further define the biological importance of *CYLD* in head and neck carcinogenesis and progression.

### *CYLD* Mutations in head and neck cancers, and other human malignancies

*CYLD* has been suggested to be a tumor suppressor gene, as supported by evidences from the first genetic susceptibility study for the *CYLD* cutaneous syndrome [1]. It is known that deleterious loss of an important tumor suppressor gene in germline settings can confer cancer predisposition in an inherited manner. A well-known comparable example is the Li–Fraumeni syndrome, a rare cancer predisposition hereditary disease caused by germline tumor protein 53 (*TP53*) mutations and the affected individuals often develop various cancers at young age. Although our current understanding of CYLD is insufficient, the very first reported case of such a cutaneous syndrome in Frances Massenger (1842) who first developed multiple scalp and face tumors, and later, multiple abdominal/peritoneal tumors reminiscent of the ones in her head and neck, and subsequently died with symptoms of cancer cachexia did suggest a potential link of the cutaneous syndrome to malignant conditions [8]. Several female family members also had a history of head and neck tumors (grandmother, mother, and sister), and breast tumors (sister), suggesting the inheritable nature of the syndrome linked to human malignancies. In fact, a recent study by Kazakov et al. reported a total of 5 patients with BSS, who were found to develop malignancies arising from pre-existing tumors in the head and neck region [26]. Further microscopic analyses of the tumors confirmed the presence of “residuum of a pre-existing benign neoplasm” indicative of in situ development of malignancies from the apparently benign lesions. A handful of malignant cases developed in patients with BSS have also been reported by others [30–49]. These malignancies included salivary gland type basal cell adenocarcinoma-like pattern, low-grade (BCAC-LG), and high grade (BCAC-HG), invasive adenocarcinomas (IACs), squamous cell carcinomas (SCCs), anaplastic neoplasms and sarcomatoid (metaplastic) carcinomas [34, 50–59].

Although it remains unclear how *CYLD* genomic aberrations precisely drive multiple head and neck tumor formation, and potentially, malignant progression, *CYLD* somatic mutations have been reported in a subset of head and neck squamous cell carcinoma (HNSCC) patients as revealed by recent WES efforts of The Cancer Genome Atlas (TCGA, USA). HNSCC is the most common type of head and neck cancer, ranking the sixth most common cancer worldwide. A total of 8 *CYLD* somatic mutations (8/279 patient cases) have been identified in primary HNSCC tumors by WES [60]. These include: F110L, V180Cfs*23, N300S, S361Lfs*47, S371*, T575S, D618A, and K680*. Among which, the S371* mutation has been found to be a hotspot germline mutation in patients with the *CYLD* cutaneous syndrome as mentioned above. Yet, the functional role of these *CYLD* mutations in HNSCC development remains unknown. Among the 8 *CYLD*-mutated HNSCC tumors, 4 were Human Papilloma virus (HPV)-negative (all smokers; age onset is 71.75 ± 3.77 years old) and the remaining 4 were HPV-positive (with 1 smoker only; age onset is 54.00 ± 6.82 years old). All HPV-negative *CYLD*-mutated tumors were also *TP53* mutated, while as expected, the HPV-positive counterparts were all *TP53* wildtype. Although all patients carrying the *CYLD*-mutated HNSCC tumors had advanced disease at the time of diagnosis [Stage III (2/8 cases) and Stage IV (6/8 cases)], the published TCGA cohort with only 8 *CYLD*-mutated cases was not able to reveal any *CYLD*-mutation and overall patient survival correlation (data not shown).

Besides the published HNSCC TCGA dataset, a recent study has identified a high incidence of *CYLD* aberrations in a rare salivary gland tumor, namely the dermal analogue tumor, which can be of sporadic or familial origins. Dermal analogue tumor is a subtype of basal cell monomorphic adenoma with remarkable histological and clinical resemblance to cylindromas. Choi et al. reported that as high as 80.9 % (17/21) of the sporadic cases, and 75 % of familial cases (9/12 tumors from two sisters) harbored LOH near the *CYLD* gene locus (16q12-13) [51]. These findings suggest that both skin adnexal tumors, which are commonly associated with the *CYLD* cutaneous syndrome, and dermal analogue tumors may share a common genetic basis, namely *CYLD* genetic alteration.

Besides HNSCC, the TCGA WES efforts also revealed other human cancers with a ≥3 % mutation rate of *CYLD*. These include (arranged in descending order of percent cases mutated in each cohort and the actual number shown in the legend; Additional file 1: Figure S1): uterine corpus endometrial carcinoma (5.2 %; 13/248 cases), lung squamous cell carcinoma (4.5 %; 8/177 cases), stomach adenocarcinoma (3.8 %; 15/395 cases) and lung adenocarcinoma (3 %; 7/230 cases). An additional 15 cancer types harbor somatic *CYLD* mutations at ~1-3 % rates. These are cancers of the skin, esophagus, colon, glioma, pancreas, liver and cervix, as well as intrahepatic cholangiocarcinoma, small cell lung cancer, large B cell lymphoma, thymoma, chromophobe renal cell carcinoma, multiple myeloma, uveal melanoma, glioblastoma (TCGA, USA; www.cbioportal.org; [61, 62]). Interestingly, two of the germline *CYLD* hotspot mutations (S371* and R758*) in *CYLD* cutaneous syndrome patients are also found in primary tumors of HNSCC, lung and stomach. Yet, the roles of these *CYLD* mutations in these solid tumors remain undetermined. It is possible that *CYLD* alterations may be involved in the tumorigenesis of many other cancers, in addition to head and neck cancers.

### CYLD signaling

Important cellular processes are known to be regulated by ubiquitination and deubiquitination of cellular proteins. Ubiquitination of a protein can determine and regulate its stability, and even its signaling functions [63]. Ubiquitins (Ubs) are small proteins (8.5 kDa) with seven lysine (K) residues (K6, K11, K27, K29, K33, K48 and K63). Ubiquitination of different K residues can serve different biological functions. For instance, K48-linked ubiquitin chains on a target protein directs the protein for proteosome degradation, while K63 links can promote protein-protein interactions and signaling activation [2].

**Fig. 2:**
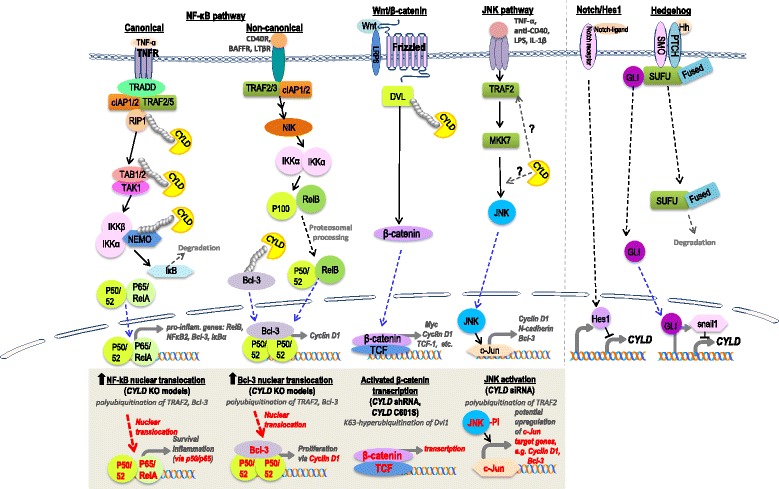
CYLD-associated signaling pathways. NF-kB, Wnt/β-catenin, and JNK pathways have been shown to be regulated by CYLD. The canonical NF-kB signaling pathway has been shown to be regulated by CYLD through deubiquitination of target substrates such as RIP1, the TAK1 complex and NEMO [2]. In the non-canonical NF-kB signaling pathway, deubiquitination of Bcl-3 by CYLD results in the inhibition of *cyclin D1* gene expression [29]. Wnt/β-catenin signaling has been shown to be regulated by CYLD, via deubiquitination of the (disheveled) DVL protein [6]. The JNK signaling pathway has been demonstrated to be regulated by CYLD activity through unknown mechanisms likely involving TRAF2 and MKK7 [7]. In addition, the Notch/Hes1 pathway and the Hedgehog signaling have been shown to regulate transcription of CYLD, via suppression of *CYLD* transcription by Hes1 and snail1, respectively [69, 70]. Blue arrows indicate nuclear translocation of the proteins. The lower grey box shows the published signaling changes and likely consequences of *CYLD* deficiencies due to *CYLD* knockout, *CYLD* silencing by siRNA or shRNA or *CYLD* mutation. Red arrows indicate that the nuclear translocation of the indicated proteins was found to be increased. Potential therapeutic targets due to *CYLD* aberrations are highlighted in red within the lower grey box

The CYLD protein has three cytoskeletal-associated protein-glycine-conserved (CAP-GLY) domains and a UCH catalytic domain with a zinc-motif [1] (Fig. 1). The CAP-GLY domains combined with proline-rich regions are responsible for microtubule and target protein binding, while the UCH domain mediates deubiquitination, and the zinc-motif allows for CYLD folding and domain interaction [1]. CYLD is highly specific for K63 ubiquitin chains [64], however has also been demonstrated to mediate K48 deubiquitination of target proteins [65]. Target proteins of CYLD include B-cell lymphoma 3 (Bcl-3), Histone-deacetylase 6 (HDAC6), Transient receptor potential cation channel A1 (TRPA1), NF-kB essential modulator (NEMO), TRAF interacting protein (TRIP), transforming growth factor-β-activated kinase 1 (TAK1), receptor-interacting protein 1 (RIP1), retinoic acid-inducible gene-1 (RIG1) and TNF-receptor associated factor (TRAF) proteins, etc. [66]. Through deubiquitination of these signaling proteins, CYLD has been shown to regulate major signaling pathways including the NF-kappaB (NF-kB) (canonical and non-canonical), Wnt/β-catenin and c-Jun NH(2)-terminal kinase (JNK) pathways (Fig. 2) [5–7, 67]. Several studies showed that the tumor suppressor CYLD inhibits NF-kB as well as the p38 MAPK pathway activation by deubiquitinating several upstream regulatory signaling molecules of these pathways, thus suppressing these signaling pathways [68]. Alternatively, CYLD has been shown to be negatively regulated by the Notch [69] and Sonic Hedgehog (Shh) [70] signaling pathways in T-cell leukemia and skin cancer, respectively (Fig. 2). As of today, among all currently identified target proteins of CYLD, many are signaling regulators of the NF-kB pathway (e.g. the TRAF proteins, NEMO, TRIP, RIP1, TAK1 and Bcl-3). Therefore, it is believed that genomic aberrations of *CYLD* may alter NF-kB signaling activity, which may also contribute to the pathophysiology of the *CYLD* cutaneous syndrome and tumor formation.

Although it is unclear if other non-NF-kB signaling pathways are potentially involved, recent evidences revealed such a possibility. *CYLD* has recently been shown to promote ciliogenesis, a process that is plausibly associated with tumorigenesis. The primary cilium is a cell surface antenna-like structure sensing chemical and mechanical signals from the environment on almost all mammalian cells. Since the formation of the primary cilium is coordinately regulated with the cell cycle progression via its connection with the centrosome, it has been hypothesized that regulators of ciliogenesis may also control cell proliferation and tissue homeostasis, and defects in primary cilium formation or function may contribute to tumorigenesis due to “non-communicative and unrestrained growth” [71–73]. In fact, in addition to this *CYLD* tumor suppressor, several key tumor suppressors and oncogenes such as the *VHL*, *PDGFR-α*, and *Shh/Patched 1* (*Shh/Ptch1*) were recently identified to regulate ciliogenesis [3, 4, 74]. Eguether et al. demonstrated that both the centrosomal localization (via interaction with a centrosomal protein CAP350) and deubiquitination activity of CYLD were required for its ciliogenic activity, independent of NF-kB [3]. Note that another NF-kB-independent and ciliogenic signaling pathway, the Shh/Ptch1 pathway, which is the most critical signaling pathway regulating cell proliferation and differentiation of basal cell carcinoma (a type of skin cancer arising from epidermal stem cell of the hair follicles) [75], has been recently identified as an upstream regulator of *CYLD* expression (Fig. 2). It remains to be investigated if this Shh/Ptch1-CYLD link is relevant for ciliogenesis as well as tumorigenesis of the skin, which can be pathologically related to this CYLD Cutaneous syndrome.

### CYLD and potential mechanisms of multiple head and neck tumor development

Although the genetic link between *CYLD* defects and the *CYLD* cutaneous syndrome has been identified, there remain many interesting questions to be answered regarding this peculiar syndrome. How do *CYLD* germline mutations give rise to “multiple” tumor formation, in particular, in the head and neck region in these patients? Furthermore, what are the molecular mechanisms underlying the progression of benign tumor lesions to malignancies in some patients?

#### Loss of CYLD links to multiple tumor development?

Almost all *CYLD* cutaneous syndrome patients do carry a germline mutation of *CYLD* which is inheritable. Interestingly, *CYLD* somatic mutations have also been identified in sporadic cases of cylindroma [1] and spiradenoma patients [76]. This evidence suggests that *CYLD* aberration is associated with the disease phenotype of multiple head and neck tumors. Thus far, *CYLD* is the only tumor suppressor gene identified to be linked with the disease. Genetically-engineered mouse models have been generated to study the function of *CYLD* in mammalian settings. A study by Massoumi et al. demonstrated that *CYLD* knockout mice (with disruption of ATG start codon) were much more susceptible to chemically-induced cutaneous squamous papilloma formation upon a single dose of 7,12-dimethybenza(a)anthracene (DMBA) followed by 12-Otetradecanoylphorbol-13-acetate (TPA) treatment [29]. All *CYLD* ^*−/−*^ mice developed skin tumors (papillomas) after 11 weeks vs. only 50–60 % of tumor incidence in *CYLD* ^*+/+*^ mice at a later time of 16 weeks. Importantly, mice with homozygous as well as heterozygous loss of *CYLD* (i.e. *CYLD* ^*−/−*^ and *CYLD* ^*+/−*^ mice) both developed multiple tumor phenotype on the skin much earlier than the *CYLD* ^*+/+*^ mice. By week 16, *CYLD* ^*−/−*^ and *CYLD* ^*+/−*^ mice harbored ~30 and 15 tumors/mouse, as compared to only 5 tumors per mouse in the *CYLD* ^*+/+*^ group. These results indicated that the loss of a single copy of *CYLD* gene was sufficient to confer a “multiple tumor phenotype” upon chemical insults in mice (although the tumor-bearing phenotype is more severe when both copies of CYLD were lost). Further, the average tumor size of papilloma developed in the *CYLD* ^*−/−*^ mice were >2.8 times of those found in the *CYLD* ^*+/+*^ mice, implicating a potential *CYLD* gene dose effect on tumor cell proliferation. Despite the fact that spontaneous tumor development was not observed in the *CYLD* ^*−/−*^ mice, loss of *CYLD* (either one or both copies) did confer a “tumor susceptible phenotype” reminiscent of patients with the *CYLD* cutaneous syndrome. It was further noted that the tumor number and size in *CYLD* ^*−/−*^ and *CYLD* ^*+/−*^ mice did grow over time after the initial DMBA/TPA insult, which is also reminiscent of the tumor characteristics reported in patients with the syndrome [1, 29]. Yet, all the tumors developed in the *CYLD* ^*−/−*^ and *CYLD* ^*+/−*^ backgrounds were hyperplastic lesions with no signs of malignancy [29]. It is likely that the loss of this *CYLD* tumor suppressor gene makes the entire epithelium of the skin highly prone to tumor initiation by chemicals or environmental insults in the “affected site”, skin in this model, thus multiple tumors can develop in this “primed soil”.

This is further supported by another *CYLD* knockout mice study, in which multiple tumors were developed in the colon of the *CYLD* ^*−/−*^ mice in a chemical-induced colitis-associated cancer (CAC) model [27], with which a DNA mutagen (azoxymethane; AOM) and an inflammation-inducing chemical (dextran sulphate sodium; DSS) were used in the drinking water to target the colon epithelium of the animals. The study demonstrated that as early as second round of DSS treatment, the *CYLD* ^*−/−*^ mice developed multiple measurable broad-based adenocarcinomas (i.e. flattened, or called sessile) in the colonic epithelium, as compared to almost no tumor in the *CYLD* ^*+/+*^ mice. In humans, it is noted that sessile polys or adenomas are pre-cancerous lesions in the colon [77]. Further investigation demonstrated that CYLD could limit inflammation and tumorigenesis by regulating ubiquitination [27]. Similar multi-tumor phenotype was also observed in a diethylnitrosamine (DEN)-induced carcinogenic liver injury model, in which significantly more, larger and multiple tumors with invasive or metastatic potential (displaying trabecular sinusoidal structures related to initial stage of invasion and metastasis in human hepatocellular carcinoma) were observed in the livers of the *CYLD* ^*−/−*^ mice as compared to that of the *CYLD* ^*+/+*^ mice [68]. The observation that multiple papillomas, colon adenocarcinomas, and liver tumors were easily induced upon treatment with chemical insults or DNA mutagens in *CYLD* knockout mice did strongly imply a generalized tumor susceptibility nature of the affected epithelium or tissue due to *CYLD* mutation or *CYLD* loss. However, it remains unclear as to why some tissues seem to develop potentially malignant tumors (e.g. liver, and colon), while some tissues tend to develop more benign tumors (e.g. skin papilloma) in vivo*.* Thus, it is important to determine if *CYLD* aberrations do confer any tissue-specific oncogenic activity in various human cancer types.

#### Why do these tumors develop predominantly in the head and neck region?

The next question is why these tumors mostly developed in the head and neck, and face of the affected individuals? The possible reason(s) may lie in the fact that these areas are always exposed to strong chemical or DNA-damaging insults. It is possible that frequent exposure to UV, a strong DNA-damaging insult can serve as a tumor inducer or potentiating agent for tumor development in the epithelium of the head and neck, and the face. It has been shown by Massoumi et al. that UV light could trigger cellular proliferation of *CYLD* ^*−/−*^ keratinocytes, as well as cyclin D1 expression [29]. The study proposed a model that in the presence of UV light and in conjunction with *CYLD* loss, Bcl-3 will translocate into the nucleus, complexed with p50 to induce cyclin D1 expression, thus cellular proliferation, while the presence of intact CYLD will inhibit Bcl-3 nuclear translocation and growth.

Another equally important possibility is the likely origin(s) of tumor from the hair stem cells as previously suggested for cylindromas [78]. As the region of head and neck, and the face harbor many stem cell -containing hair follicles in the sebaceous and sweat glands, *CYLD* genetic aberrations may affect the proliferation control, or inflammatory status of the stem cell niches, thus resulting in predominant head and neck tumor formation. Evidence for this can be noted as these tumors never grow from the hair-less parts of the body (e.g. the palms and soles), but only in the hairy parts of the body. It is also possible that hair follicle stem cells that harbor *CYLD* alterations may acquire additional genetic changes over one’s lifetime thus resulting in tumor formation. However, since the origin of these tumors of the *CYLD* cutaneous syndrome patients is still of debate, this hypothesis remains to be proven. Another possibility that remains to be proven is that, maybe, CYLD is specifically and functionally associated with developmental control or growth regulation of the head and neck or hair follicles in humans. Thus, germline defects of *CYLD* in patients with the *CYLD* cutaneous syndrome are mainly presented with head and neck tumors or tumors in regions with lots of hair follicles.

As *CYLD* somatic mutations occur in HNSCC tumors, and *CYLD* aberrations seem to be the key genetic driver for multiple head and neck tumor formation in patients with this cutaneous syndrome, an unanswered question is whether *CYLD* aberration alone is sufficient to directly drive head and neck tumor formation. Do additional genetic or chemical insults associated with head and neck carcinogenesis, such as smoking, drinking, or HPV infection, promote tumorigenesis in *CYLD-*mutated head and neck cancers? Is the immune system involved as well, since CYLD is also implicated in the regulation of immunity? All these questions remain to be addressed.

## Conclusions

The genetics of the *CYLD* cutaneous syndrome underlies the formation of multiple tumors in the head and neck epithelium. Current treatments are limited, except for repeated surgical removal of the tumors when needed. Inhibition of NF-kB signaling can potentially be a treatment option. Yet, a prior clinical trial on the topical use of salicylic acid showed some efficacies in some affected individuals only (2/12 cases) [79]. A recent study showed that *CYLD* mutations can cause activation of the tropomyosin kinase (TRK) signaling in tumors of affected individuals [80]. Further, inhibition of TRK signaling in *CYLD*-mutant tumor models demonstrated the potential efficacies of TRK targeting. Thus TRK inhibitors can be a potential treatment strategy for these patients. It is important to understand more about the genetics and biology of these *CYLD*-mutant tumors, which may point to new treatment or prevention of these disfiguring tumors. Further understanding of the role of CYLD in head and neck epithelial biology may also identify mechanisms of tumorigenesis and progression of head and neck cancers, as well as other human malignancies.

## Additional file

Additional file 1: Figure S1. Graph showing the mutation frequencies of *CYLD* gene in major cancer types. Data were extracted from the cBioPortal database (www.cbioportal.org; dated 3rd August, 2016). The *CYLD* mutation frequencies of 15 most updated TCGA Provisional cancer cohorts, and five other important cancer types with *CYLD* mutation rates of >1–3 % rates were shown, with actual number of mutated cases shown in this legend. *Abbreviations:* Uterine (TCGA Provisional): Uterine Corpus Endometrial Carcinoma 13/248 cases (5.2 %), Lung squ (TCGA Provisional): Lung Squamous Cell Carcinoma 8/177 cases (4.5 %), Stomach (TCGA Provisional): Stomach Adenocarcinoma 15/395 cases (3.8 %), Lung adeno (TCGA Provisional): Lung Adenocarcinoma 7/230 cases (3 %), Head & neck (TCGA Provisional): Head and Neck Squamous Cell Carcinoma 15/512 cases (2.9 %), Cholangiocarcinoma (JHU, 2013): Intrahepatic Cholangiocarcinoma 1/40 (2.5 %), Small Cell Lung (JHU, 2012): Small Cell Lung Cancer 1/42 (2.4 %), Melanoma (TCGA Provisional): Skin Cutaneous Melanoma 8/368 cases (2.2 %), Esophagus (TCGA Provisional): Esophageal Carcinoma 4/185 cases (2.2 %), DLBC (TCGA Provisional): Lymphoid Neoplasm Diffuse Large B-cell Lymphoma 1/48 case (2.1 %), Colorectal (TCGA Provisional): Colorectal Adenocarcinoma 4/223 cases (1.8 %), Glioma (UCSF, 2014): Low-Grade Gliomas 1/61 (1.6 %), Thymoma (TCGA Provisional): Thymoma 2/123 cases (1.6 %), chRCC (TCGA Provisional): Kidney Chromophobe 1/66 case (1.5 %), MM (Broad, 2014): Multiple Myeloma 3/205 (1.5 %), Pancreas (TCGA Provisional): Pancreatic Adenocarcinoma 2/150 cases (1.3 %), Uveal melanoma (TCGA Provisional): Uveal melanoma 1/80 case (1.3 %), GBM (TCGA, 2008): Glioblastoma 1/91 (1.1 %), Liver (TCGA Provisional): Liver Hepatocellular Carcinoma 4/373 cases (1.1 %), Cervical (TCGA Provisional): Cervical Squamous Cell Carcinoma & Endocervical Adenocarcinoma 2/194 cases (1 %). (PPTX 77 kb)

## Abbreviations

DMBA: 7,12-dimethybenza(a)anthracene
TPA: 12-O-tetradecanoylphorbol-13-acetate
BCAC: Basal cell adenocarcinoma
BCAC-HG: Basal cell adenocarcinoma-like pattern high grade
BCAC-LG: Basal cell adenocarcinoma-like pattern low-grade
BCC: Basal cell carcinoma
Bcl-3: B-cell lymphoma 3
BSS: Brooke-Spiegler Syndrome
CAP350: Centrosome-Associated Protein 350
cIAP1/2: Cellular inhibitor of apoptosis 1 and 2
JNK: c-Jun NH(2)-terminal kinase
CCD: Clear cell differentiation
CYLD: Cylindromatosis
CAP-GLY: Cytoskeletal-associated proteinglycine-conserved
DUB: Deubiquitinating
DSS: Dextran sulphate sodium
DEN: Diethylnitrosamine
Dvl: Dishevelled
FC: Familial Cylindromatosis
FISH: Fluorescence in-situ hybridization
HNSCC: Head & neck squamous cell carcinoma
Hes1: Hes Family BHLH Transcription Factor 1
HDAC6: Histone-deacetylase 6
HPV: Human Papilloma virus
T: Individual tumor
IACs: Invasive adenocarcinomas
IKKα/IKKβ: IkB Kinase α and β
LOH: Loss of heterozygosity
LRP6: Low-density lipoprotein receptor-related protein 6
LEF/TCF: Lymphoid enhancer factor/T-cell factor
K: Lysine
MEFs: Mouse embryonic fibroblasts
Md: Mild
MKK7: Mitogen-Activated Protein Kinase 7
MFT1: Multiple Familial Trichoepithelioma 1
NGS: Next generation sequencing
NIK: NF-kappa-B inducing kinase
NEMO: NF-kB essential modulator
NF-kB: Nuclear Factor-kB
PTCH1: Patched 1
PDGFR-α: Platelet-derived growth factor receptor
RIP1: Receptor-interacting protein 1
RB1: Retinoblastoma 1
RIG1: Retinoic acid-inducible gene-1
S: Severe
SMO: Smoothened
snail1: Snail family transcriptional repressor 1
Shh: Sonic Hedgehog
Shh/Ptch1: Shh/Patched 1
SCCs: Squamous cell carcinomas
SUFU: Suppressor of Fused
TAB1: TGF-beta activated kinase 1
TAK1: TGF-β-activated kinase 1
TRAF: TNF receptor associate factor
TRADD: TNFRSF1AAssociated Via Death Domain
TRIP: TRAF interacting protein
TRPA1: Transient receptor potential cation channel A1
TRK: Tropomyosin kinase
TNFR: Tumor necrosis factor receptor
TNF-α: Tumor necrosis factor-α
TP53: Tumor protein 53
UCH: Ubiquitin C-terminal Hydrolase
Ubs: Ubiquitins
VEGF-A: Vascular endothelial growth factor-A
VS: Very severe
WES: Whole-exome sequencing
