# Supplementary figures and images for: The cylindromatosis (CYLD) gene and head and neck tumorigenesis
Source: Cancers Head Neck. 2016 Sep 8;1:10. doi: 10.1186/s41199-016-0012-y (PMC6460526; doi:10.1186/s41199-016-0012-y)

## Slide 1
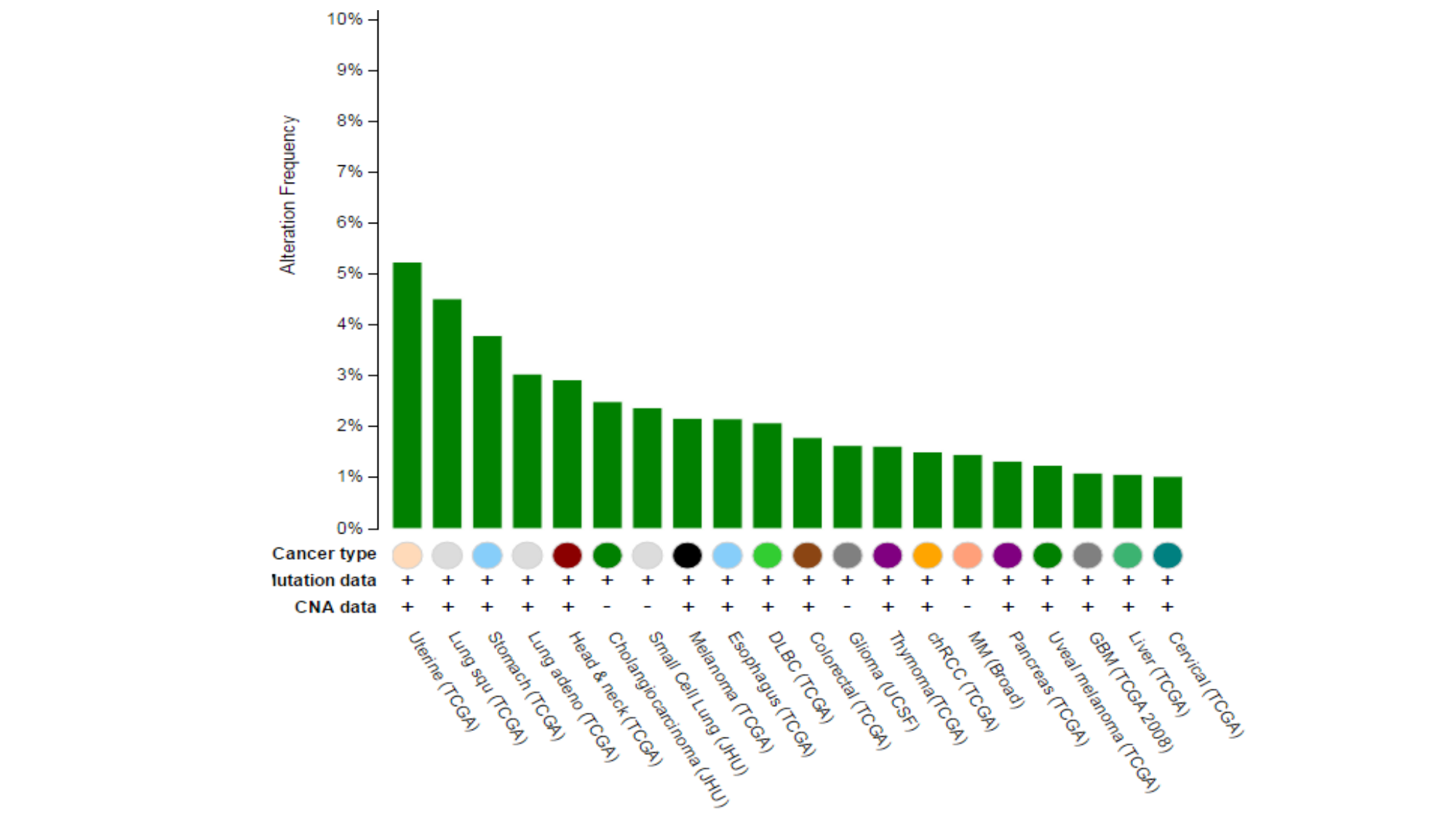

Supplement: Supplementary file 1 — Graph showing the mutation frequencies of CYLD gene in major cancer types. Data were extracted from the cBioPortal database (www.cbioportal.org; dated 3rd August, 2016). The CYLD mutation frequencies of 15 most updated TCGA Provisional cancer cohorts, and five other important cancer types with CYLD mutation rates of >1–3 % rates were shown, with actual number of mutated cases shown in this legend. Abbreviations: Uterine (TCGA Provisional): Uterine Corpus Endometrial Carcinoma 13/248 cases (5.2 %), Lung squ (TCGA Provisional): Lung Squamous Cell Carcinoma 8/177 cases (4.5 %), Stomach (TCGA Provisional): Stomach Adenocarcinoma 15/395 cases (3.8 %), Lung adeno (TCGA Provisional): Lung Adenocarcinoma 7/230 cases (3 %), Head & neck (TCGA Provisional): Head and Neck Squamous Cell Carcinoma 15/512 cases (2.9 %), Cholangiocarcinoma (JHU, 2013): Intrahepatic Cholangiocarcinoma 1/40 (2.5 %), Small Cell Lung (JHU, 2012): Small Cell Lung Cancer 1/42 (2.4 %), Melanoma (TCGA Provisional): Skin Cutaneous Melanoma 8/368 cases (2.2 %), Esophagus (TCGA Provisional): Esophageal Carcinoma 4/185 cases (2.2 %), DLBC (TCGA Provisional): Lymphoid Neoplasm Diffuse Large B-cell Lymphoma 1/48 case (2.1 %), Colorectal (TCGA Provisional): Colorectal Adenocarcinoma 4/223 cases (1.8 %), Glioma (UCSF, 2014): Low-Grade Gliomas 1/61 (1.6 %), Thymoma (TCGA Provisional): Thymoma 2/123 cases (1.6 %), chRCC (TCGA Provisional): Kidney Chromophobe 1/66 case (1.5 %), MM (Broad, 2014): Multiple Myeloma 3/205 (1.5 %), Pancreas (TCGA Provisional): Pancreatic Adenocarcinoma 2/150 cases (1.3 %), Uveal melanoma (TCGA Provisional): Uveal melanoma 1/80 case (1.3 %), GBM (TCGA, 2008): Glioblastoma 1/91 (1.1 %), Liver (TCGA Provisional): Liver Hepatocellular Carcinoma 4/373 cases (1.1 %), Cervical (TCGA Provisional): Cervical Squamous Cell Carcinoma & Endocervical Adenocarcinoma 2/194 cases (1 %). (PPTX 77 kb) [file 41199_2016_12_MOESM1_ESM.pptx]
